# On the Lesions and Pathological Phenomena Caused by the Presence of Lumbrici in the Biliary Ducts

**Published:** 1859-01

**Authors:** 


*On the Lesions and Pathological Phenomena caused by the Presence of Lumbrici in the Biliary Ducts.* By Dr. E. A. Bonfils. (Archives Générales, June, 1858.)

After combating Cruveilhier's opinion, that intestinal worms can be introduced into the biliary ducts only after death or during the death struggle, Dr. Bonfils analyses the 23 cases which he has collected, in which lumbrici ere discovered in the ductis communis choledochus, in the gall-bladder, or in the hepatic duct; in 2 cases the lumbrici were perfectly fresh and still living; in 1 the worm was dead and slightly altered, was of a pure white, and softened; in 1, reported by M. Forget, a lumbricus occupying the ductus communis and the ductus hepaticus was perfectly fresh, while another occupying an abscess in the right lobe of the liver was softened and macerated, evidently having been long dead; in 1 case a lumbricus formed the nucleus of a biliary calculus. The symptoms varied much in the different cases, but the author considers that the presence of the following circumstances justifies the conclusion that we have to deal with the presence of a lumbricus in the biliary ducts: the sudden appearance of morbid phenomena, without appreciable moral or physical causes, of considerable intensity, characterized by very violent pain, combined with deep color of the skin, vomiting, &c., similar to the symptoms accompanying calculus in the biliary passages; a rapid disappearance of all phenomena on the discharge of the worm; the concurrence of these symptoms, unassociated with general colicky pains (*coliques extérieures*), are regarded by the author as indicative of a lumbricus being the foreign body which has entered the biliary ducts, and having thus arrested the passage of the bile.—*Ib.*

